# Reassessment of Poststroke Dysphagia in Rehabilitation Facility Results in Reduction in Diet Restrictions

**DOI:** 10.3390/jcm10081714

**Published:** 2021-04-15

**Authors:** Anna Maria Pekacka-Egli, Radoslaw Kazmierski, Dietmar Lutz, Katarzyna Pekacka-Falkowska, Adam Maszczyk, Wolfram Windisch, Marc Spielmanns

**Affiliations:** 1 Department for Pulmonary Medicine and Sleep Medicine, Zürcher RehaZentren, Klinik Wald, 8636 Wald, Switzerland; Marc.spielmanns@zhreha.ch; 2 Department for Neurology and Neurorehabilitation, Zürcher RehaZentren, Klinik Wald, 8636 Wald, Switzerland; Dietmar.lutz@zhreha.ch; 3 Department for Neurology and Cerebrovascular Disorders, Poznan University of Medical Sciences, 61701 Poznan, Poland; rkazmierski@ump.edu.pl; 4 Department for History and Philosophy of Medicine, Poznan University of Medical Sciences, 61701 Poznan, Poland; pekackafalkowska@ump.edu.pl; 5 Department for Methodology, Statistics, and Informatics Systems, Institute of Sport Science, Academy of Physical Education in Katowice, 40065 Katowice, Poland; a.maszczyk@awf.katowice.pl; 6 Department for Pulmonary Medicine, Faculty of Health, University Witten-Herdecke, 58455 Witten, Germany; windischw@kliniken-koeln.de; 7 Department of Pneumology, Cologne Merheim Hospital Kliniken der Stadt Koeln GmbH, 51109 Koeln, Germany

**Keywords:** dysphagia, reassessment, diet, stroke, poststroke care

## Abstract

Background: Dysphagia assessment in postacute stroke patients can decrease the incidence of complications like malnutrition, dehydration, and aspiration pneumonia. It also helps to avoid unnecessary diet restrictions. The aim of this study is to verify if regular reassessment of dysphagia would change the diet management of postacute stroke patients in rehabilitation settings. Methods: This single-center retrospective study included 63 patients referred to an inpatient neurological rehabilitation center between 2018–2019. A standardized clinical swallowing evaluation and Fiberoptic Endoscopic Evaluation of Swallowing (FEES) were performed. Diet level according to Functional Oral Intake Scale (FOIS) was evaluated. As the primary endpoint, the FOIS values based on diagnostic procedures were assessed at hospital discharge, rehabilitation admission, and after FEES. Results: 19 women (30%) and 44 men (70%), with a mean age of 75 y (SD ± 10.08), were enrolled. The intergroup ANOVA revealed significant differences (*p* < 0.001) between dietary prescriptions in an acute care setting and following clinical and endoscopic reassessment in the rehabilitation center. Diet recommendations changed in 41 of 63 (65%) enrolled patients (*p* < 0.001). Conclusion: Instrumental diagnostic by FEES during the early convalescence period of stroke patients leads to clinically relevant changes to diet restrictions and lower rates of pneumonia. Our findings underline the need for regular and qualitative dysphagia diagnostics in stroke patients participating in neurological rehabilitation.

## 1. Introduction

The total number of prevalent strokes, deaths, and disability-adjusted life years due to stroke has increased steadily worldwide from 1990, reaching 101 million prevalent stroke survivors, 6.55 million deaths from stroke, and 143 million disability-adjusted life years due to stroke in 2019 [1]. Dysphagia occurs in about 50% of these patients, with an incidence increase depending on whether bedside or instrumental diagnosis is performed [2,3]. Dysphagia can result in aspiration and might lead to aspiration pneumonia as well as malnutrition, dehydration, and increased mortality [4,5]. Poststroke dysphagia is also associated with an increased length of hospitalization and higher hospital costs [6]. Research has shown lower incidences of aspiration pneumonia in facilities using screening programs to detect aspiration or dysphagia [4,7]. 

Dysphagia can be assessed using various methods, including bedside swallowing tests or a formal instrumental assessment. Up to now, no gold standard dysphagia assessment protocol has been defined and implemented [8]. There is a variety of clinical guidelines and recommendations available for the management of dysphagia [9,10,11]. The ESPEN Guideline Clinical Nutrition in Neurology recommends that stroke patients undergo a formalized swallow test prior to any oral intake. Dietary adjustments should only be ordered after an assessment of swallowing function, including the assessment of aspiration risk. A standardized protocol (clinical and, if possible, instrumental) should be used by professionals that are trained and experienced in the assessment and management of dysphagia. Re-evaluation of the swallowing act should be performed at regular intervals until swallowing function is regained [2,11]. 

Videofluoroscopic swallowing study (VFSS) and fiberoptic endoscopic evaluation of swallowing (FEES) are complimentary modalities. According to international organizations nowadays, both methods should, ideally, be available [9]. Recent research has shown that the implementation of FEES in the diagnostic regime leads to a revision of diet recommendations in nearly 70% of neurological patients [12]. Not every hospital has a stroke unit, employs speech and language pathologists (SLP), or has qualified personal for performing dysphagia screening or instrumental assessment. These circumstances are the cause for very restrictive diet modifications, ensuing malnutrition, insufficient fluid intake, and lower quality of life of stroke survivors [13,14]. 

The aim of this study is to verify if the reassessment of dysphagia and, in particular, FEES would change the diet management of poststroke patients and lead to clinically relevant changes in diet restrictions in the rehabilitation setting.

We hypothesize that the reassessment of dysphagia during the early convalescence period of stroke patients may lead to significant changes in diet restrictions.

## 2. Materials and Methods

The patient data and results of the assessments were sourced for analysis from our clinic information system (Phoenix^TM^, CompuGroup Medical AG, 3007 Bern, Switzerland). We used RehaTIS^TM^ (Softsolution, International AG, 15,830 Lahti, Finland) to record and manage the individual rehabilitation process of each participant, including all therapies and procedures. All patients gave written informed general consent at admission. The local ethics committee approved the study protocol (Kantonale Ethikkommision Zürich, BASEC-No. 2020-02148).

### 2.1. Participants

We retrospectively analyzed the records of 620 patients with an ICD-10-CM Code: I60–I69 diagnosis (cerebrovascular diseases) who had been referred for neurological inpatient rehabilitation to Zürcher RehaZentren, Klinik Wald, Switzerland from stroke units and acute wards of various regional hospitals between January 2018 and December 2019. Sixty-three patients met the study inclusion criteria and were enrolled. The mean period from stroke onset to presentation in our rehabilitation facility was 2.5 weeks (SD ± 7 days). The inclusion criteria were as follows:

(1) the first diagnosis of stroke and
(2) stable medical condition.

We excluded patients with

(1) known history of swallowing difficulties due to previous strokes (*n* = 69),
(2) presence of neurological conditions other than stroke, which could lead to dysphagia (*n* = 19),
(3) no referral for SLP intervention (*n* = 220),
(4) referral for SLP intervention for reasons other than dysphagia (*n* = 168),
(5) patients who required a change in oral diet due to reasons other than dysphagia (i.e., poor nutrition, poor dental status) (*n* = 28)
(6) data missing in the medical records’ prior dysphagia assessments (*n* = 21),
(7) transferred back to a hospital before completed diagnostics (*n* = 32).

The baseline characteristic data analyzed included age, sex, stroke type, type of dysphagia assessment prior to rehabilitation, type of diet (including feeding tube dependency before admission), occurrence of pneumonia, and length of rehabilitation stay.

### 2.2. Study Design 

**Figure 1:**
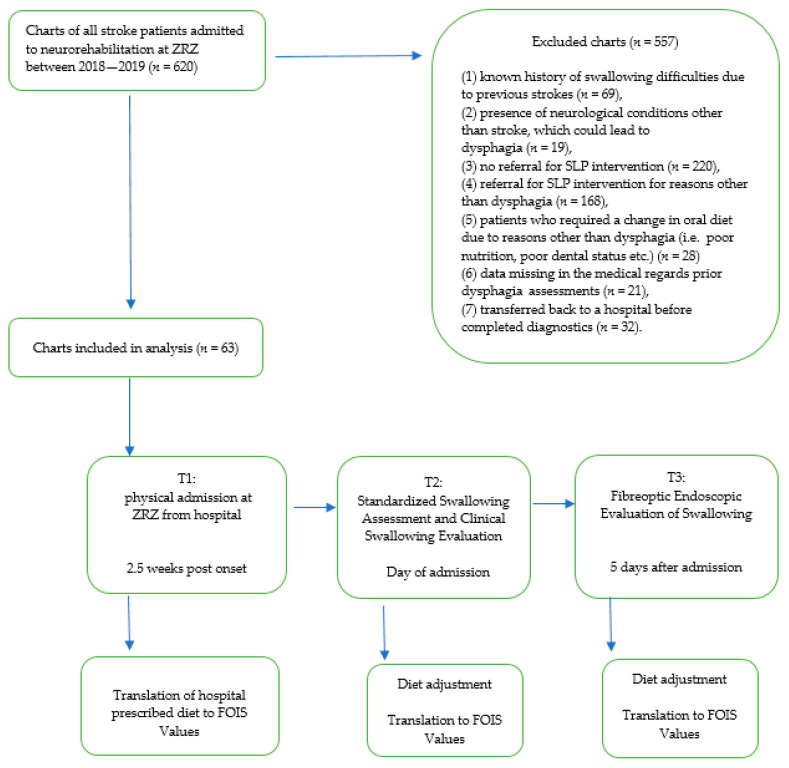
Chart review process. Abbreviations: ZRZ, Zurcher RehaZentren; SLP, speech and language pathologist; FOIS, Functional Oral Intake Scale.

The chart review process is depicted in Figure 1.

On the day of admission, the dietary levels assigned in the acute care institution (T1) were translated to Functional Oral Intake Scale (FOIS) [15] values. Within 5 days after admission for rehabilitation, all patients were assessed for dysphagia. Following the clinical (T2) and instrumental assessments (T3), the individual’s swallowing ability in relation to FOIS was compared with the initially prescribed dietary level. 

### 2.3. Procedures

#### 2.3.1. Screening Procedures for Presence of Dysphagia and Referral to SLP

Within the first 6 h, patients admitted to the rehabilitation facility underwent specific screening procedures by nurses, physicians, physiotherapists, and occupational therapists. On that basis and the results of the clinical diagnosis, the so-called “admission coordination report” is issued. It defines the goal of treatment to be attained, the limitations of the patients, the results of swallow screening by nurses, and the need for other types of therapy (e.g., neuropsychology, speech and language therapy, music therapy). Dysphagia was considered present when

(1) listed as a medical diagnosis,
(2) confirmed by previous diagnostic procedures documented in the patient’s records,
(3) diet status prescribed in acute care,
(4) stated by staff in the admission-coordination-report.

#### 2.3.2. Clinical Dysphagia Assessment 

After referral to SLT, the risk of dysphagia was reassessed with the Standardized Swallowing Assessment (SSA) using a binary present/absent scoring [16]. This method contains items relevant but not related to swallowing, as well as an evaluation of water swallowing and a test meal (in our institution, applesauce, banana, and bread are fixed menu items). 

In the next step, the Clinical Swallowing Evaluation (CSE) was performed according to the in-house protocol based on the screening protocol for neurogenic dysphagia [17,18]. This method contains the following items: testing of vigilance, ability to control and swallow saliva, and patients’ ability to swallow semisolid, liquid, and solid consistency. The aspiration predictors were recorded during the examination using binary present/absent scoring. An aspiration risk was considered present if two or more of the following six predictors had been scored as present: (1) dysarthria, (2) dysphonia, (3) abnormal gag reflex, (4) abnormal volitional cough, (5) voice change after swallowing, and (6) cough after swallowing.

#### 2.3.3. Instrumental Assessment 

FEES was performed as a standard diagnostic procedure for all patients suspected of dysphagia. An experienced neurologist and one or two certified SLPs carried out the FEES protocol. The examination was recorded and subjected to a video analysis (rpSzene^®^, Rehder & Partner Company, Methfesselstraße 74, 20,257 Hamburg, Germany). Due to institutional protocol, the neurologist supervised the analysis and authorized the final FEES report.

Our in-house FEES protocol and implementation are based on the recommendations by Langmore [19]. The FEES protocol consists of the examination of the anatomical structures involved in swallowing, secretion rating, and swallow examination. Exactly defined amounts of pudding—thick consistency (1 teaspoon = 5 mL), water (1 teaspoon = 5 mL, 1 sip = 10 mL) and solid food (cookie; 5 g) were tested. Each consistency was offered 3 times. After classifying the consistencies as uncertain, the corresponding consistency was abandoned.

The results were analyzed using the Rosenbeks Penetration–Aspiration Scale (PAS), with the highest PAS score of all tested consistencies recorded. The results were recorded and then classified as follows: (1) PAS I–II: normal–mild (no aspiration, penetration with clearing) (2) PAS III–V: moderate (penetrations), (3) PAS VI–VIII: severe (aspirations), and (4) PAS VIII: silent aspiration [20].

#### 2.3.4. Oral Intake Status

The type of diet was determined for each patient after the clinical and instrumental swallowing examination. The dietary levels (including the dietary levels assigned by the acute care institution) were translated into Functional Oral Intake Scale (FOIS) values. 

FOIS was used to assess the oral intake of the patients classified by an ordinal rating scale, including seven tries. Levels 1–3 describe tube dependency, where Level 1 describes patients with no oral intake, and Levels 2–3 describe tube-dependent patients. Levels 4–7 describe full oralization, where Levels 4–6 are for patients who have not yet reached oral diet expansion, and Level 7 is for patients who have fully reached diet expansion [15]. 

#### 2.3.5. Functional Independence Measurement (FIM)

To measure the change in functional limitations of patients during rehabilitation, the Functional Independence Measurement (FIM) is performed on admission and before discharge. FIM is an 18-item measurement tool that explores the severity of an individual’s physical and psychological disability, especially in rehabilitation patients [21].

### 2.4. Statistical Analysis

All variables were expressed as mean ± standard deviation (SD). Before using a parametric test, the assumption of normality was verified using the Kolmogorov–Smirnov test. The distributions of all variables were normal or close to normal. The numbers of quality data for analyzing groups were obtained using an analysis of the contingency table. Student *t*-test was used to determine differences in baseline characteristics between the groups of patients with and without diet changes. 

One-way ANOVA was used, with significance set at *p* < 0.05, to determine intergroup statistically significant differences between PAS scores for FOIS variables prior to rehabilitation, at admission, and after instrumental diagnostic. When appropriate, a Tukey post hoc test was used to compare selected data, and the effect of each test was calculated to determine the significance of the results. The effect size (η2) was classified according to Hopkins as 0.01—small, 0.06—medium, and 0.14—large. Values of *p* < 0.05 were considered significant. The remaining analyses were performed using STATISTICA ((Stat Soft, Inc., Tulsa, USA; 2018) version 12).

## 3. Results

The study group consisted of 63 persons, including 19 women and 44 men, with a mean age of 75 (SD ± 10.08) years. The baseline characteristics and differences in patients with and without a change in diet type are summarized in Table 1. 

At the time of transfer (T1), 34 patients were fully tube-dependent (FOIS 1). Seven were tube-dependent with some oral intake (FOIS 2–3). Seventeen patients were not tube-dependent but restricted in their oral diet (FOIS 4–6); five patients had no restrictions in oral intake. The mean FOIS value at T1 was 2.59 (SD ± 2.08).

The initial dysphagia screening (T2) was positive in 58 of 63 patients. In 57 patients, two or more predictors of aspiration were recorded. Only 6 patients presented with one or no clinical indicator but were referred for further diagnostic workup due to clinical suspicion of dysphagia. Based on these results, we changed the diet recommendations in 27 patients, with lower dietary restrictions in 19 patients and increased restrictions in 8 patients. In 36 patients, we did not make any diet modifications; 27 patients were still tube-dependent. The mean FOIS value at T2 was 2.79 (SD ± 1.81). 

**Figure 2:**
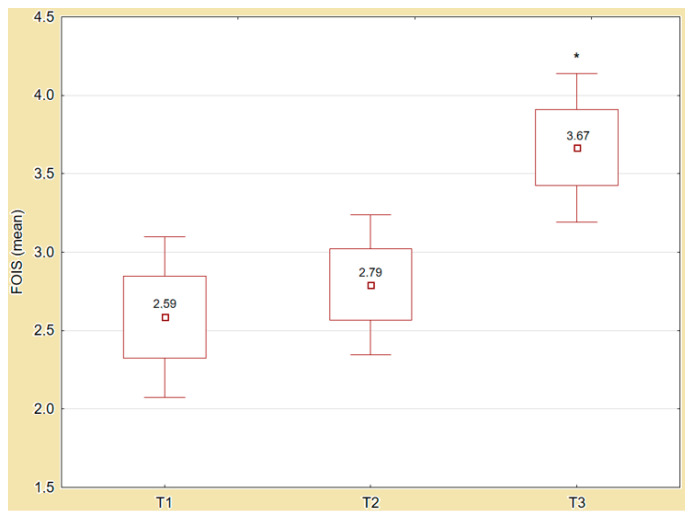
Changes in FOIS values (T1–T3). Abbreviations: T1, hospital discharge; T2, rehabilitation admission; T3, after FEES; * statistically significant value with *p* = 0.001. The intergroup ANOVA revealed statistically significant differences in diet recommendations at T1, T2, and at T3 based on Functional Oral Intake Scale values (*p* = 0.001). In the next step, the posthoc test and effect size analysis were done. Table 2 presents the results of the posthoc tests and differential values in the aspects for FOIS at time of procedure. Analysis of the effect size revealed a large effect for FOIS (η2 = 0.190) after FEES at T3.

On average, all 63 patients underwent FEES examination (T3) five days following the first dietary prescription. FEES identified 21 patients with penetration episodes (PAS III–V). Aspiration (PAS VI–VIII) was found in 38 patients, including 24 patients with silent aspiration (PAS VIII). Due to the FEES results, a diet modification was required in 41 patients, with a lowering of restrictions in 38 cases. In three patients, we increased restrictions, and one patient additionally received a nasogastric tube to protect him from aspiration. In 12 cases, no oral intake was recommended. The mean FOIS value at T3 was 3.67 (SD ± 1.92). Figure 2 illustrates the changes to the FOIS values at the different times of the stay/diagnostic workup (T1–T3). 

The intergroup ANOVA revealed statistically significant differences between PAS scores for FOIS variables at T1, T2, and T3 (*p* = 0.001). The contingency tables analysis revealed 25 individuals with PAS I–V and 38 with PAS VI–VIII, including 24 individuals only with PAS VIII. Posthoc tests revealed statistically significant differences between aspiration (PAS VI–VIII and VIII) vs. normal (PAS I–II) and penetration values (PAS III–V) for FOIS at T1, T2, and T3. A statistically significant increase was observed for FOIS at T3 in reference to FOIS at T1 and T2 in all aspects of PAS I–II, III–V, VI–VIII, and VIII groups (Table 3).

Furthermore, we found differences in baseline characteristics in patients with adjustment of diet. In this group, rates of pneumonia were significantly lower than in patients without diet change (8 vs. 16, with *p* = 0.034). We also noted significantly higher general FIM scores at discharge in those patients (mean 74.5 (SD ±17.4) vs. 68.5 (SD ±13.15); *p* = 0.031). 

The length of stay was only minimally shorter in the patient group with diet change than in patients without diet adjustment (38 days (SD ± 19.5) vs. 39 days (SD ± 20.5), *p* = 0.780). However, patients with restrictive adjustment of diet had a longer rehabilitation stay of 42.5 days (SD ± 22.5) vs. 39 days (SD ± 18) (*p* = 0.337).

## 4. Discussion

The aim of this study is to verify if the reassessment of dysphagia and, in particular, FEES would change the diet management of poststroke patients and lead to clinically relevant changes in diet restrictions in the rehabilitation setting.

The results of the study illustrate that a comprehensive reassessment of poststroke dysphagia in the rehabilitation setting contributed significantly to clinically relevant changes in the diet restrictions of 65% of our cohort. 

The necessity for dysphagia reassessment as a routine has not yet been established, although national and international guidelines recommend an assessment of dysphagia in stroke survivors in acute care [2,9]. Our study provides similar findings for the rehabilitation setting. We see the need for the re-evaluation of dysphagia in postacute care for a change in dietary prescriptions for patients after stroke; however, due to the retrospective character of this limited study, further prospective research is recommended. 

Only in a minority of the patients’ transfer records (40%), documentation of the initial dysphagia assessment of the acute care facility was included. In the majority (60%), the dysphagia assessment in acute care was not mentioned. Heckert et al. reported comparable rates in 2009. In their study population, the diagnosis of dysphagia was mentioned in only 36% of transfer records [22].

In this study, all patients with suspected dysphagia were assessed using clinical and instrumental diagnostics. The results showed that a standardized swallowing assessment was positive for the presence of dysphagia in 92% of cases, which is comparable to previously published data [23]. However, in our study, the high rate of dysphagia might be interpreted by considering the inclusion criteria, which resulted in the exclusion of a large part of the sample. In the clinical swallowing study, 90% of our patients were found to be at risk of dysphagia and aspiration by establishing two or more clinical predictors of aspiration. This is also comparable to previously published data [24]. Daniels et al. reported that the identification of two or more clinical predictors can predict aspiration risk in patients with swallowing disorders [25]. Based on the diagnostic results, we changed the dietary recommendations for 43% of patients; 27 patients were still tube-dependent. This can be explained by the low sensitivity of the clinical swallowing examination. Due to the imprecise conclusions of the clinical examination, a more restrictive diet is generally prescribed [26].

According to Warnecke et al. [27], the accuracy of a clinical swallowing evaluation in patients with acute stroke is inadequate, and the diagnostic yield of instrumental diagnostics is more accurate. As already mentioned, the clinical dysphagia examination alone shows insufficient sensitivity and specificity. For this reason, an instrumental diagnosis is required to objectify the swallowing process. Appropriate diet prescription decisions can be made based on the instrumental diagnostic results. This statement also seems to apply to patients in the postacute phase of stroke.

Based on the FEES results, a significant change in diet occurred in 65% of the patient cohort in this study, with diet restrictions being decreased in 60% and increased in 5% of patients. These results are supported by a recent German hospital study by Braun et al. [28]. In 72% of their study cohort, the FEES investigation found relevant dysphagia, which led to an adjustment of diet. In addition, the authors observed a lower rate of pneumonia and a better functional outcome on leaving the clinic in the patients with an adapted diet. In another study, Bax et al. found a significant reduction in the rate of pneumonia in the patient cohort diagnosed with FEES [29]. These patients had both longer hospital stays and longer nil-by-mouth periods but were significantly more likely to leave the hospital on a full oral diet.

Our study shows comparable results. We observed a statistically significant increase in FOIS values after the FEES examination (T3) compared to the FOIS values on discharge (T1) and after the clinical swallowing examination (T2) in all aspects of penetration and aspiration episodes. The present study also shows significantly lower rates of pneumonia, a significantly better general FIM score on discharge, and a shorter length of stay in patients with dietary changes.

According to Cohen et al., pneumonia prevention does not only include the early detection of swallowing disorders [28]. Both therapeutic and dietary interventions to reduce aspiration volume and frequency, as well as procedures to reduce the pathogenicity of aspiration, improve larynx sensitivity and respiratory protection mechanisms, as well as cough, and promote cortical plasticity to restore the swallowing function [30]. A combination of different interventions for protection from aspiration pneumonia is possible, e.g., implementation of swallowing screening, a change in the consistency of food and drink, early mobilization of patients, and, if necessary, a timely introduction of enteral nutrition.

At the time of transfer from the hospital, 54% of our patients were tube-dependent. The study by Teuschl et al. showed similar results, where 60% of cases of pneumonia were recorded in patients without oral intake [31].

Brogan et al. argued that there is a relationship between pneumonia and long waiting times for clinical swallowing tests in patients, as these waiting times have been synonymous with oral abstinence [32]. A similar observation was made by Brady et al. [33]. According to their assessment by the UK National Stroke Registry, waiting time for admission screening is linearly related to the risk of pneumonia. An absolute increase in the incidence of pneumonia of 1% per waiting day was found. 

In our cohort, the time span between admission and introduction of instrumental diagnostics was 5 days (mean). In patients with an adjustment of diet, we also observed significantly lower rates of pneumonia compared to patients without diet change. That assumes that the implementation of the diagnostic approach might have prevented the occurrence of pneumonia.

Some limitations exist in our study. It is a retrospective cohort study performed at a single center, with unavoidable biases and limitations. Moreover, the sample size was small, and we lacked a validated tool for the screening of dysphagia. To validate and expand our findings, future prospective research in this field is needed.

## 5. Conclusions

Instrumental diagnostics by FEES during the early convalescence period of stroke patients lead to clinically relevant changes to the diet and lower rates of pneumonia. Our findings underline the need for regular and qualitative dysphagia diagnostics during stroke rehabilitation.

## Figures and Tables

**Table 1:** Baseline characteristics of the study group (*n* = 63) and differences in patients with and without a change in diet type.

Characteristics	Total Cohort (*n* = 63)	NCOD (*n* = 22)	COD (*n* = 41)	*p* (NCOD vs. COD)
Age	75.25 (10.08)	73.73 (11.49)	76.27 (8.32)	*p* > 0.05
Male	44	12	32	***p* < 0.05**
Female	19	10	9	*p* > 0.05
Stroke type				
				***p* < 0.05**
Ischemic	53	18	35	*p* > 0.05
Hemorrhage	10	4	6	
FIM points at admission	49.32 (21.91)	42.72 (18.91)	49.37 (22.08)	*p* > 0.05
FIM points at discharge	71.44 (31.10)	68.50 (13.15)	74.50 (17.40)	***p* < 0.05**
Dysphagia assessment prior to NR				
Cinical diagnostic only	8	3	5	*p* > 0.05
Instrumental diagnostic only	0	0	0	*p* > 0.05
Clinical and instrumental diagnostic	11	5	6	*p* > 0.05
Assessment not specified.				
No documentation of	6	2	4	*p* > 0.05
dysphagia assessment	38	12	26	***p* < 0.05**
Feeding tube dependency at admission to NR				
PEG-Tube	27	10	17	*p* > 0.05
NG-Tube	14	8	6	*p* > 0.05
Time from admission to clinical assessment in days	0 (0.82)	0 (0.815)	0 (0.825)	*p* > 0.05
Time from admission to first FEES in days	5 (3.95)	4 (1.98)	5 (2.01)	*p* > 0.05
Length of stay in rehabilitation in days	38 (20)	39 (20.5)	38 (19.5)	*p* > 0.05
Pneumonia	24	8	16	***p* < 0.05**

Abbreviations: NCOD, no change in diet group; COD, change in diet group; (SD), standard deviation; FIM, Functional Independence Measure; FEES, Fibreoptic Endoscopic Evaluation of Swallowing; PEG, percutaneous endoscopic gastrostomy; NG, nasogastric; NR, neurorehabilitation. Statistical significance is marked by bold values.

**Table 2:** Results of posthoc tests for the Functional Oral Intake Scale prior to rehabilitation, after clinical assessment, and after FEES diagnostics.

Functional Oral Intake Scale			
	T1	T2	T3
	Differential values 2.59	Differential values 2.79	Differential values 3.67
T1		0.210	**0.001**
T2	0.210		**0.001**
T3	**0.001**	**0.001**	

Abbreviations: T1, hospital discharge; T2, rehabilitation admission; T3, after FEES; Statistical significance is marked by bold values.

**Table 3:** Results of posthoc tests for the Functional Oral Intake Scale in the aspects of the Penetration–Aspiration Scale I–II, III–V, VI–VIII, and VIII groups.

Functional Oral Intake Scale at T1				
Groups	**PAS I–II**	**PAS III–V**	**PAS VI–VIII**	**PAS VIII**
	Differential values 4.000	Differential values 3.875	Differential values 1.902	Differential values 1.815
PAS I–II		0.999	0.103	0.094
PAS III–V	0.999		**0.001**	**0.001**
PAS VI–VIII	0.103	**0.001**		0.997
PAS VIII	0.094	**0.001**	0.997	
**Functional Oral Intake Scale at T2**				
Groups	Differential values 5.500	Differential values 4.083	Differential values 2.195	Differential values 2.000
PAS I–II		0.391	**0.001**	**0.001**
PAS III–V	0.391		**0.001**	**0.001**
PAS VI–VIII	**0.001**	**0.001**		0.964
PAS VIII	**0.001**	**0.001**	0.964	
**Functional Oral Intake Scale at T3**				
Groups	Differential values 7.000	Differential values 5.250	Differential values 2.902	Differential values 2.482
PAS I–II		0.243	**0.001**	**0.001**
PAS III–V	0.243		**0.001**	**0.001**
PAS VI–VIII	**0.001**	**0.001**		0.758
PAS VIII	**0.001**	**0.001**	0.758	

Abbreviations: T1, hospital discharge; T2, rehabilitation admission; T3, after FEES; PAS I–II, no aspiration, penetration with clearing; PAS III–V, penetration episodes; PAS VI–VIII, severe aspirations; PAS VIII, silent aspiration; Statistical significance is marked by bold values.

## Data Availability

Data supporting the reported results can be accessed by corresponding with the authors.
